# Advancing Concrete Mix Proportion through Hybrid Intelligence: A Multi-Objective Optimization Approach

**DOI:** 10.3390/ma16196448

**Published:** 2023-09-28

**Authors:** Feixiang Chen, Wangyang Xu, Qing Wen, Guozhi Zhang, Liuliu Xu, Dingqiang Fan, Rui Yu

**Affiliations:** 1 CCCC Second Harbor Engineering Company Ltd., Wuhan 430070, China; 2 Key Laboratory of Large-Span Bridge Construction Technology, Wuhan 430070, China; 3 CCCC Highway Bridge National Engineering Research Centre Co., Ltd., Wuhan 430070, China; 4 State Key Laboratory of Silicate Materials for Architectures, Wuhan University of Technology, Wuhan 430070, Chinar.yu@whut.edu.cn (R.Y.); 5 Department of Civil and Environmental Engineering, The Hong Kong Polytechnic University, Kowloon, Hong Kong 999077, China

**Keywords:** concrete, mix design, multi-objective optimization, artificial neural network (ANN), genetic algorithm (GA), Scipy library

## Abstract

Concrete mixture design has been a key focus in concrete research. This study presents a new method for concrete mixture design by combining artificial neural networks (ANN), genetic algorithms (GA), and Scipy libraries for hybrid intelligent modeling. This method enables the prediction of concrete mechanical properties and the optimization of mix proportions with single or multi-objective goals. The GA is used to optimize the structure and weight parameters of ANN to improve prediction accuracy and generalization ability (R^2^ > 0.95, RMSE and MAE < 10). Then, the Scipy library combined with GA-ANN is used for the multi-objective optimization of concrete mix proportions to balance the compressive strength and costs of concrete. Moreover, an AI-based concrete mix proportion design system is developed, utilizing a user-friendly GUI to meet specific strength requirements and adapt to practical needs. This system enhances optimization design capabilities and sets the stage for future advancements. Overall, this study focuses on optimizing concrete mixture design using hybrid intelligent modeling and multi-objective optimization, which contributes to providing a novel and practical solution for improving the efficiency and accuracy of concrete mixture design in the construction industry.

## 1. Introduction

Concrete is a typical construction material generally composed of cement, sand, aggregates, and water. Characterized by strong plasticity and easy construction, concrete has been the most widely used material in various constructions, such as encompassing residential buildings, bridges, tunnels, and roads [1,2]. The structural integrity and reliability of these constructions significantly depend on the strength of the concrete [3]. Consequently, it is imperative to assess concrete strength within a specified time frame after curing, which is costly, particularly in challenging environmental conditions. Meanwhile, concrete strength testing often entails prolonged measurement periods, posing a challenge due to the inherent conflict between strength requirements and economic considerations. Enhancing concrete strength necessitates optimizing the aggregate ratio, controlling the water/cement ratio, and adding the appropriate amount of chemical mixture, and during the process of developing the concrete mix proportions that meet the design requirements, the cost of concrete will rise. Therefore, in practical engineering scenarios, it becomes imperative to strike a good balance between concrete strength and economic constraints.

The conventional approach for concrete mixture design relies on a trial-and-error methodology, which is not only time consuming but also cost intensive [4,5]. Particularly in intricate experimental settings, the design process becomes further challenging and expensive. The conventional methods typically rely on empirical formulas and test data, primarily focusing on meeting a single performance criterion, such as strength or workability [6]. However, when multiple objectives need to be addressed simultaneously, the traditional approach requires many experiments to achieve a balance among these objectives. Furthermore, to regulate the performance of concrete mixtures, researchers incorporated multiple admixtures and additives. This further increases the difficulty of the mixture design. As a result, researchers have proposed alternative methods such as mathematical optimization, design of experiments (DOE), statistical mixed design (SMD) techniques [7,8], and D-optimal design (DOD). For example, Abhilash et al. [7] combined the DOE approach to design and analyze different mixtures with the goal of avoiding error variance and identifying influential factors, and, this way, represent the experiments on the axial behavior of concrete-filled steel tube columns. Fan et al. [9] developed a new eco-friendly ultra-high performance concrete (UHPC) using a numerical optimization method based on the established DOD model. However, these methods may overlook the intricacies of cement hydration reactions, potentially resulting in unsuitable mixtures. These limitations hinder the rapid analysis of key factors affecting concrete performance and the ability to meet mix ratio requirements in practical applications. Therefore, there is a need for a novel method that surpasses existing empirical and statistical models, providing more efficient solutions for concrete mix design.

In recent years, artificial intelligence (AI) has experienced rapid development, which has enabled efficient processing and analysis of large datasets while also simulating human thinking and behavior [10]. Machine learning (ML) is a typical component of AI, which has witnessed significant growth in the materials and engineering frontier, as well as in the field of concrete. Indeed, this data-driven model-based approach has been used for the multiscale characterization and design of different types of concrete engineering materials and structures [11]. Among the different types of ML-based cases, the artificial neural network (ANN) is currently one of the most used methods. For example, Gao et al. [12] ML models, including both traditional single models and new ensemble learning models, to accurately assess and predict the frost resistance development of waste rubber in complex environments. Biswas et al. [13] proposed an integrated approach using the Runge–Kutta optimizer and ANN to predict the compressive strength of SCC based on the percentage of supplementary cementitious materials replacing cement. The obtained correlation coefficient (R^2^ = 0.92) indicates that machine learning models can accurately estimate the compressive strength of SCC. In addition, Xue et al. [14] proposed a method of establishing a multiscale model based on the ANN to predict the macroscopic mechanical properties of heterogeneous concrete materials by using microstructural data.

Although ANNs have many advantages, they still possess certain limitations. If the amount of data is insufficient, it may cause the network to overfit or underfit, thus affecting the prediction accuracy [15]. Despite the flaws mentioned above, ANNs are attractive to researchers due to their ability to mine data and solve complex prediction and classification problems. Hence, some methods were proposed to solve these problems, such as genetic algorithms (GA), particle swarm algorithms, simulated annealing algorithms, and ant colony algorithms. The goal of these algorithms is to improve their performance and prediction accuracy by modifying the weights and structure of the neural network. Using GA to optimize ANN can not only enhance their performance and generalization ability but also improve their global search ability and interpretability [16]. GA can also improve scalability, which means they can be extended to multi-objective optimization and multiple types of ANN structure and weight parameter optimization problems. For example, Zhang et al. [17] proposed a neural network model based on GA, which can be used to estimate the compressive strength of rubber concrete with ultrasonic pulse velocity. The results showed that GA-ANN (Genetic Algorithm–Artificial Neural Network) had the highest accuracy compared to the other regression models and was proven to be reasonable for precise estimation. Moreover, combining GA with the Scipy optimization algorithm makes it easier to find the minimum or maximum value of the objective function [18]. The Scipy library also supports constrained optimization algorithms, where some additional constraints need to be considered in addition to finding the minimum or maximum values [18]. These constraints can be equality constraints or inequality constraints, used to limit the value range of the optimization variables or to satisfy certain conditions.

Based on these considerations, this study proposes the utilization of machine learning (ML) techniques for performance prediction and multi-objective optimization of concrete materials. Specifically, a reliable dataset is collected from real engineering projects, and the correlation between data features and independent variables is analyzed. Subsequently, a GA-ANN model is established to predict the compressive strength of concrete, and the accuracy of this model is thoroughly evaluated. Finally, a multi-objective search algorithm (Scipy library) is employed to achieve the optimization of the concrete mixture within the desired strength range and under cost constraints.

In conclusion, this study combines ANN and GA to optimize the structure and weight parameters of the ANN model, which improves prediction accuracy and generalization. Scipy library is utilized for multi-objective optimization of concrete mixtures, contributing to making trade-offs between different goals and obtaining concrete mixtures that meet both strength requirements and cost considerations. Last but not least, based on a hybrid intelligent modeling approach, a GUI software V1.0 is developed, which not only predicts the compressive strength of concrete but also provides guidance for engineers and researchers in the concrete mixture design process. By incorporating AI technology, the software enhances the efficiency and accuracy of concrete mixture design, allowing for the optimization of various performance characteristics.

## 2. Methodology

### 2.1. Data Collection

The dataset used in this study comes from the CCCC Second Harbor Engineering Company LTD and contains 500 data. Eight input variables are considered in the data set, cemented materials including cement (OPC), fly ash (F), and admixtures (AD), fine aggregate (F-A), coarse aggregate (C-A), in addition to water (W) and superplasticizer (SP). The compressive strength and cost of the concrete prepared are obtained experimentally, with results that are taken as the output value.

Moreover, Table 1 presents the characteristics of cement, mineral powder, and fly ash, while Table 2 displays the basic characteristics of the aggregate. The experimental cement used was Ordinary Portland Cement P.II 42.5. The 28-day activity index of mineral powder and fly ash was 95–122% and 71–97%, respectively. The fine aggregate includes machine-made sand and natural sand. As indicated in Table 1, the fineness modulus of machine-made sand and natural sand was 2.4–3.3 and 2.5–3.1, respectively. The mud content of natural sand was between 1.2 and 2.6. Table 2 shows that the coarse aggregate comprised three types of crushed stone particle sizes, namely 5–10 mm, 10–20 mm, and 16–31.5 mm, with corresponding void ratios ranging from 37 to 45%, 38 to 45%, and 42 to 44%, respectively. The high-efficiency water reducer is a high-performance polycarboxylate-based superplasticizer with an average solid content of 20.8%.

During the sample mixture compaction and curing method, a standard procedure was followed. The curing temperature was maintained at 20 ± 2 °C, and the relative humidity was kept at no less than 95%. The strength was tested using a 150 × 150 × 150 mm test block and in accordance with the standard (GB/T 50081-2019) [19]. The workability of freshly poured concrete was also tested according to the standard (GB/T 50080-2016) [20]. Based on relevant specifications and actual engineering requirements, the general collapse range was determined to be between 40 and 220 mm.

In this dataset, Table 3 lists the parameter range, standard deviation, and mean value, and the non-normally distributed datasets. In the context of ML models, the parameter range defines the permissible range of input variables, while the standard deviation quantifies the degree of dispersion among the variable data. On the other hand, the average reflects the central tendency of the variable data. These statistical measures play a crucial role in understanding and defining the characteristics of the input variables for ML models. Therefore, the provided information holds significant importance as it enhances our understanding of the patterns and characteristics inherent in the dataset.

### 2.2. Preliminary Information of Algorithms

#### 2.2.1. ANN Model

Artificial neural networks (ANNs) are a typical ML model inspired by the structure and function of the human brain [21]. ANNs consist of a network of interconnected nodes, or neurons, which receive inputs, process information through mathematical operations, and produce output signals [22,23]. Each neuron is connected to other neurons via weighted connections, which determine the strength of the signal between them. The output of each neuron is determined by applying an activation function to the weighted sum of its inputs. The most used activation functions include the sigmoid function, the hyperbolic tangent function, and the rectified linear unit (ReLU) function.

The mathematical operations performed via ANNs are based on linear algebra, matrix multiplication, and calculus [24]. In the process of initializing an ANN, the weights connecting the neurons are typically assigned random values. The ANN is then trained using an optimization algorithm, such as gradient descent or backpropagation. The objective of training is to minimize a cost function, which quantifies the discrepancy between the predicted outputs generated by the ANN and the actual outputs. The training process involves repeatedly adjusting the weights to improve the accuracy of the predictions. The performance of ANNs can be evaluated using various metrics, such as mean squared error (MSE), root mean squared error (RMSE), mean absolute error (MAE), and coefficient of determination (R^2^) [24]. These metrics provide a quantitative measure of the accuracy and reliability of the predictions.

The ANN is composed of multiple neuronal models connected according to certain rules. The commonly used neuronal model is the fully connected neuron named ‘M-P neuron model’. The multi-layer perceptron structure and general formula for MP neurons in an ANN can be expressed in Equation (1) [25].

(1) $$y_{j}=f\left(\sum_{i=1}^{n}x_{i}w_{ij}+x_{0}w_{0j}\right)=f\left(\sum_{i=1}^{n}x_{i}w_{ij}-\theta \right)$$

where $\left\{x_{1}\ldots x_{n}\right\}$ is the input of neurons, $\left\{w_{1j}\ldots w_{nj}\right\}$ is the weight that corresponds to the input, and θ is bias.

ReLU function is a popular activation function in ML models [23]. Compared with sigmoid function and tanh function, it has the following advantages: when the input is positive, there is no gradient saturation problem, and the computation is much faster. Only linear relationships exist in the ReLU function, so it is calculated faster than sigmoid and tanh [23,24].

The sigmoid function is defined as Equation (2):

(2) $$f\left(z\right)=\frac{1}{1+{\mathrm{e}}^{-z}}$$

The hyperbolic tangent function is defined as Equation (3):

(3) $$f(x)=tanh(x)=\frac{2}{1+e^{-2x}}-1$$

The ReLU function is defined as Equation (4):

(4) $$\sigma (x)=\{\begin{array}{cc} max(0,x) & ,x>=0\\ 0 & ,x<0 \end{array}$$

In ANN models, the weights between neurons are commonly initialized randomly. The ANN is then trained using an optimization algorithm, such as gradient descent or backpropagation, with the aim of minimizing a cost function. This cost function quantifies the difference between the predicted outputs generated by the ANN and the actual outputs observed in the training data. The optimization algorithm iteratively adjusts the weights in order to improve the accuracy of the predictions and reduce the cost function to its minimum value [26,27]. The cost function is often defined as mean squared error (MSE). The training process involves repeatedly adjusting the weights to improve the accuracy of the predictions. This is carried out by calculating the gradient of the cost function with respect to the weights and updating the weights in the opposite direction of the gradient, multiplied by a learning rate.

The test dataset is used to verify the predictive ability of the optimized artificial neural network model. To make a more comprehensive and better comparison, some statistical error analysis parameters are used as criteria for evaluating the model [28]. Important statistical parameters are used in this study, including mean squared error (MSE), root mean square error (RMSE), mean absolute error (MAE), and Pearson coefficient of determination (R^2^) [25]. These parameters can be calculated as follows in Equations (5)–(8) [25]:

(5) $$MSE=\frac{1}{n}{\sum_{n}^{1}\left|x_{i}-y_{i}\right|}^{2}$$

(6) $$RMSE=\sqrt{\frac{1}{n}{\sum_{n}^{1}\left|x_{i}-y_{i}\right|}^{2}}$$

(7) $$MAE=\frac{1}{n}\sum_{i=1}^{n}\left|x_{i}-y_{i}\right|$$

(8) $$R^{2}=1-(\frac{\sum_{i=1}^{n}(x_{i}-y_{i}{)}^{2}}{\sum_{i=1}^{n}(x_{i}-\bar{x}{)}^{2}})$$

where n is the total number of datasets, $x_{i}$ and $y_{i}$ represents the predicted value and the target value, respectively, and $\bar{x}$ is the average of the target value.

#### 2.2.2. Algorithm Structure of GA-ANN

**Figure 1:**
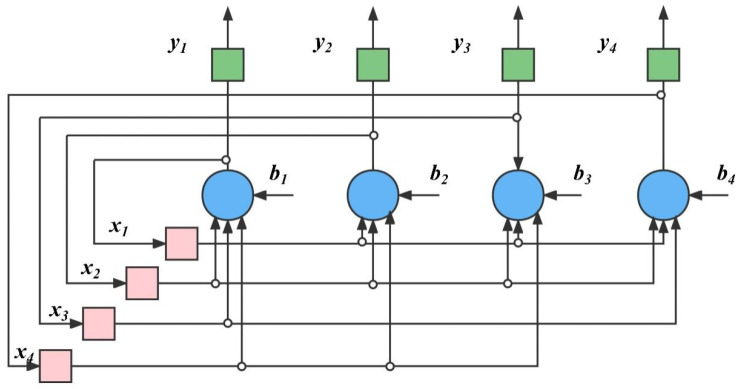
Topology diagram of ANN.

GA originated from computer simulation studies conducted on biological systems is a stochastic global search optimization method [16]. It simulates phenomena such as replication, crossover, and variation occurring in natural selection and inheritance [11]. Through random selection, crossover, and variant manipulation, the initial population is transformed into a group of individuals. This iterative process allows for the evolution of the group towards better regions in the search space. Generation after generation, the individuals adapt and converge, ultimately resulting in a high-quality solution to the problem. The combination of GA and ANN (GA-ANN) is a hybrid method used to optimize the parameters of the ANN to improve their performance. ANNs provide search space and use genetic algorithms to find the best solution by adjusting the weights and bias to achieve lower error rates. The core idea of GA-ANN is that crossing the weights of two good neural networks produces a better neural network. The reason for pleasantly surprised effectiveness of GA is that it does not directly optimize the ANN network structure, which can result in extremely diverse outcomes [11]. The optimization process includes the following steps (Figure 1):

Step 1: Collect the dataset and perform data mining.

The dataset includes input vectors and output vectors. The input vectors in this article are OPC (*x*_1_), FA (*x*_2_), MP (*x*_3_), Admixture (*x*_4_), F-A (*x*_5_), C-A (*x*_6_), Water (*x*_7_), and SP (*x*_8_), while the output vector is compressive strength (T1) and a correlation equation between input and output vectors is established. To show the relationship between input and output vectors, a heatmap can be used. By combining knowledge from materials science, it is possible to explain the reasons for the influence on concrete performance.

Step 2: Determine the network structure of the ANN model.

Firstly, the topology of the neural network is determined. In this article, the topology is a feedback-type neural network structure in which each neuron simultaneously feeds its own output signal back to other neurons as an input signal, and it takes a while to work to reach stability. Hopfield neural networks (HNN) belong to this type [29], shown in Figure 1. The number of neurons, layers, inputs, and outputs for the network are also determined.

(9) $$y_{j}(t+1)=f[\sum_{i=1}^{n}\omega_{\overset{ˉ}{y}}\chi_{i}(t)]-T_{j}$$

where $\chi_{i}$ represents multiple inputs, and ω represents the weight of each input, which simulates the excitement and inhibition of protrusions in biological neurons. Sigma represents the summation and integration of all input signals, f is the activation function, and y is the output.

Then, determine the parameters of individual neurons, weights, and biases. According to Equation (1), the output signals of each neuron can be calculated [29], as seen in Equation (9).

(10) $$\Delta W_{i}=\eta \times \delta \times o$$

where $\Delta W_{i}$ means the adjusted weight, η means the training rate, δ represents the local gradient of layer, and o demonstrates the output of the input neurons.

**Figure 2:**
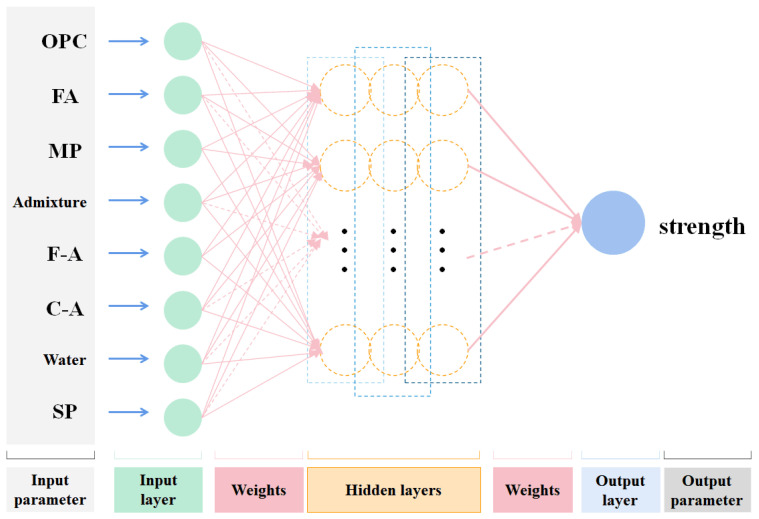
The multilayer perceptron ANN.

Finally, determine the multi-layer perceptron (MLP), including the input layer (*x_i_*), hidden layer, and output layer (*y*), as specifically illustrated in Figure 2. The error between the output value and the actual value can be calculated and passed back to the network to adjust the weights [29], summarized as Equation (10).

**Figure 3:**
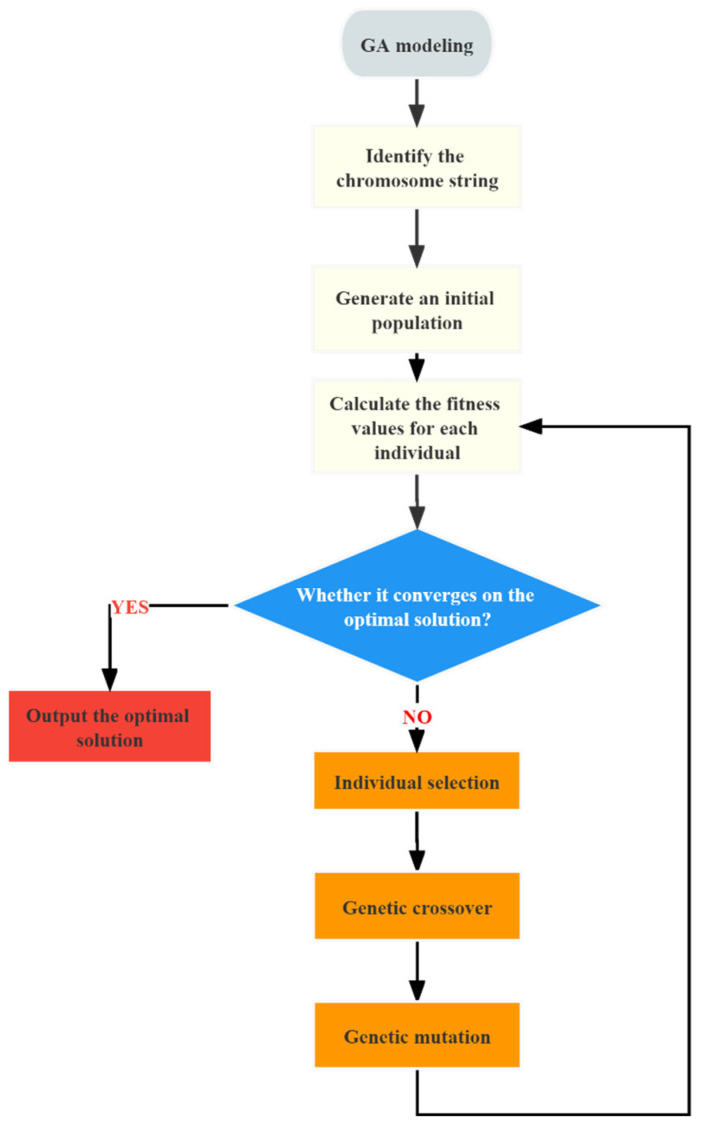
Process flowchart of GA.

Step 3: Choosing optimization algorithm—GA (process flowchart shown in Figure 3).

(a) Establish the fitness function. One characteristic of GA is its ability to obtain search information using only the objective function of the problem at hand. The objective function is reflected in the evaluation of individual fitness for minimization problems [29], which is shown in Equation (11).

(11) $$F=C_{\alpha }-f$$

where F represents the fitness function, f means objective function, and C is a constant.

(b) Individual selection. GA uses selection decisions to choose superior individuals and eliminate inferior ones. High-fitness individuals are then passed on to the next generation. The proportionate fitness scaling method is a classic selection decision method that calculates an individual’s selection probability based on its fitness value [28], shown in Equation (12).

(12) $$p_{i}=\frac{f_{i}}{\sum_{j=1}^{n}f_{j}}$$

where f is the fitness value of individual in population, and n is the population size.

(c) Genetic crossover. Crossover is the process of replacing and recombining parts of genes from two parent individuals to generate a new individual. When designing the crossover operator, one must consider the individual encoding design. Simulated binary crossover (SBX) is a frequently used crossover operator in continuous optimization methods, and the selected gene changes followed Equations (13) and (14) [28].

(13) $$y_{i}^{1}=0.5[(1-\beta )x_{i}^{1}+(1+\beta )x_{i}^{2}]$$

(14) $$\beta (u)=\{\begin{array}{c} (2u)\frac{1}{n_{c}+1},\frac{4u}{n_{c}}u\leq 0.5\\ (2(1-u))\frac{1}{n_{c}+1},\;or \end{array}$$

where x is the parent individual, y is the way the selected gene changes, and u is a uniformly distributed random number in region of [0, 1].

(d) Genetic mutation. Introducing mutations not only improves the local search ability of the GA but also maintains diversity among individuals in the population. The simplest mutation method is uniform mutation, which replaces the original gene value of each gene in the individual with a uniformly distributed random number within a certain range [28], with a small probability, which is shown in Equation (15).

(15) $$x_{k}^{\prime }=U_{min}^{\;}+r(U_{max}^{\;}-U_{min}^{\;})$$

where r is a uniformly distributed random number in region of [0, 1].

**Figure 4:**
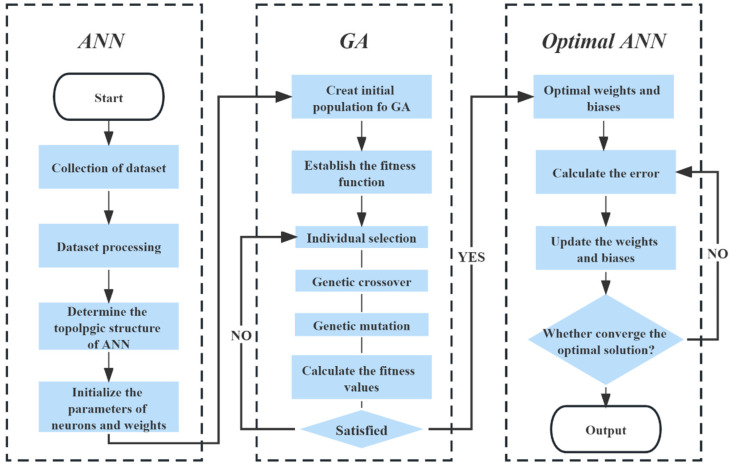
Flowchart of GA-ANN.

Step 4: Optimize ANN with GA (shown in Figure 4).

Machine learning models are trained and evaluated. Training an ML model is an iterative process that requires constantly adjusting the parameters of model to improve its accuracy and efficiency. Evaluating an ML model involves measuring its performance with a test dataset. If the performance of the model is not satisfactory, it is necessary to go back to previous steps with adjustments until the desired results are achieved.

#### 2.2.3. Multi-Optimization with Scipy

Optimization algorithms systematically explore potential solutions to approximate the optimal solution, making them invaluable in various domains, such as engineering, computer science, physics, and economics [11,29]. Their primary objective is to maximize or minimize an objective function that represents the problem’s objective. The significance of optimization algorithms lies in their ability to expedite the search for optimal solutions and handle large-scale problems. Complex problem solutions often entail extensive computations, but optimization algorithms facilitate faster and superior outcomes, thus expediting the overall process. Single-objective optimization and multi-objective optimization are two pivotal branches of the optimization algorithm. In single-objective optimization, the algorithm focuses on maximizing or minimizing a solitary objective function [30]. For instance, in the realm of concrete, a single-objective optimization problem could involve seeking a manufacturing solution with the lowest cost. Conversely, multi-objective optimization entails the maximization or minimization of multiple objective functions, often conflicting with one another [31,32]. For example, achieving maximum concrete strength while minimizing costs presents a typical multi-objective optimization scenario.

Scipy provides a variety of optimization algorithms that can be used to solve different types of optimization problems. Based on Scipy, the optimization algorithms are used to find the optimal solution for a given problem. The problem can be either to maximize or minimize an objective function. Furthermore, ‘scipy.optimize.minimize’ is a function available in the ‘scipy’ library that offers a unified interface for minimizing different optimization algorithms, such as nonlinear least squares, nonlinear programming, and root finding, which are defined as Equations (16)–(20) [33]. It is capable of finding the minimum of a scalar function of one or more variables, considering constraints [11]. The function requires a function to minimize, an initial guess for the optimal solution, and several optimization parameters as input. The output is an object that contains the optimal solution and other information, such as the number of function evaluations and the status of the optimization.

(16) $$\underset{\beta }{min\;}\sum_{i=1}^{n}[y_{i}-f(x_{i},\beta ){]}^{2}$$

In nonlinear least squares, where the non-linear model is $f(x_{i},\beta )$, where x is the independent variable, x is the dependent variable, and β is the model parameter.

(17) $$minimize\;\;f(x)=\sum_{j=1}^{n}c_{j}x_{j}$$

(18) $$\begin{array}{cc} subject\;to & s.t.:\{\begin{array}{cc} h_{j}(x)\leq 0, & j=1,\ldots ,q\\ g_{i}(x)=0, & i=1,\ldots ,p \end{array} \end{array}$$

In nonlinear programming, where $x=[x_{1},\ldots ,x_{n}{]}^{T}$ means decision variables, f(x) represents objective functions, $h_{j}(x)$ and $g_{j}(x)$ are constraints.

(19) $$f(x)\approx f(x_{n})+f^{\prime }(x_{n})(x-x_{n})$$

(20) $$x_{n+1}=x_{n}-\frac{f(x_{n})}{f^{\prime }(x_{n})}$$

Root finding is the process of finding the zero of a function by approximating the zero point repeatedly. For instance, the Newton method uses the Taylor series expansion to estimate the value of the function f(x) at point $x_{n}$, sets f(x) to zero, and obtains the next approximate solution $x_{n+1}$ (seen in Equations (19) and (20)). This process is repeated until convergence to the zero point.

The Scipy library includes a function called Scipy.optimize, which uses an iterative-based optimization algorithm to find the minimum or maximum value of an objective function. The algorithm works by determining the gradient and Hessian matrix of the objective function in the direction of the step matrix of the variable vector in the next step. The gradient and Hessian matrices represent the first and second derivatives of the objective function at a specific point [31,32].

## 3. Results and Discussion

For evaluating the influence of various components of raw materials on the mechanical properties of concrete, the third part conducts a correlation analysis on the complex relationships between various raw material parameter variables based on statistical analysis methods. Then, a GA-ANN model is established to predict the compressive strength of concrete and compared with the ANN model. Finally, combined with the Scipy algorithm, a multi-objective optimization design is conducted to obtain a mix ratio that meets both the high strength and low economic cost of concrete. At the same time, an expert evaluation system is established to assist in concrete design.

### 3.1. Prediction of Compression Strength Based on GA-ANN

#### 3.1.1. Dataset Processing and Evaluation

The construction of the ANN model relies on datasets comprising input and output labels [11]. In this paper, the experimental data utilized for training and testing the ANN model is sourced from factory experiments. This selection ensures the consistency of raw materials and mitigates errors stemming from variations in material sources and experimental environments. Moreover, obtaining accurate training models for input data at varying scales proves challenging. Hence, the preprocessing of raw data assumes crucial significance in the modeling process.

**Figure 5:**
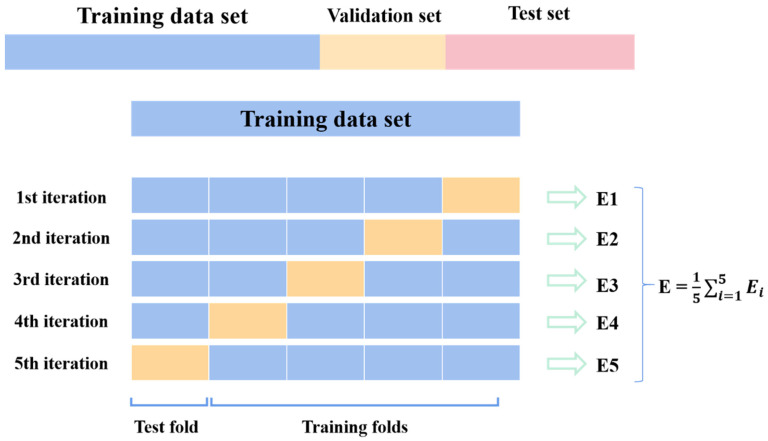
Division of database.

The preprocessing of the dataset generally consists of two parts: one is the reasonable division of the dataset, and the other is the normalization or standardization of the dataset. The dataset is divided mainly to complete the training and testing of the model, and the common allocation rates for training, validation, and testing are 60%, 20%, and 20%, as shown in Figure 5. The training set is used to train the model, and the validation set is aimed to verify its accuracy. Finally, the trained model is applied to predict the testing set and analyze and compare the predicted value with the true value. The min-max scaling and Z-score standardization of input and output data are designed to prevent premature saturation of coverage and hidden nodes between numbers of different sizes, which are shown in Equations (21) and (22).

(21) $$X_{norm}=\frac{X-X_{min}}{X_{max}-X_{min}}$$

where $X_{norm}$ represents the normalized data, X is the raw data, $X_{max}$ and $X_{min}$ are, respectively, the maximum and minimum values of the original data set, especially in min-max scaling.

(22) $$z=\frac{x-\mu }{\sigma }$$

where μ and σ are the mean and the variance of the original dataset, especially in Z-score standardization.

#### 3.1.2. Prediction and Verification of GA-ANN Model

GA can optimize the structure and weight parameters of ANN, improving their prediction accuracy and generalization ability [21,22]. GA is a search algorithm that can find optimal solutions in complex search spaces. Compared to traditional gradient descent methods, GA is more suitable for nonlinear, non-convex, multimodal, and high-dimensional optimization problems. Additionally, GA can consider multiple objectives simultaneously, finding a balance point between them and improving ANN performance [23,34]. Another advantage of GA is that it maintains population diversity, preventing premature convergence to local optimal solutions.

When optimizing ANN, selecting the right network parameters is crucial. These parameters can have a significant impact on the performance of the network and prediction accuracy. This study employed a feedback-type topology structure consisting of eight input vectors and one output vector for the prediction of compressive strength. The established ANN has four hidden layers, each containing 50 neurons, with an epoch of 200 times, a learning rate of 0.001, a training batch size of 2, a testing batch size of 40, and a ReLU activation function, which has been one of the latest and most commonly used functions [33,35]. Commonly, Metaheuristics was used to adjust the network parameters because the structure of the network was the connection mode and the number of connections between the neural layers, which were very complex.

**Figure 6:**
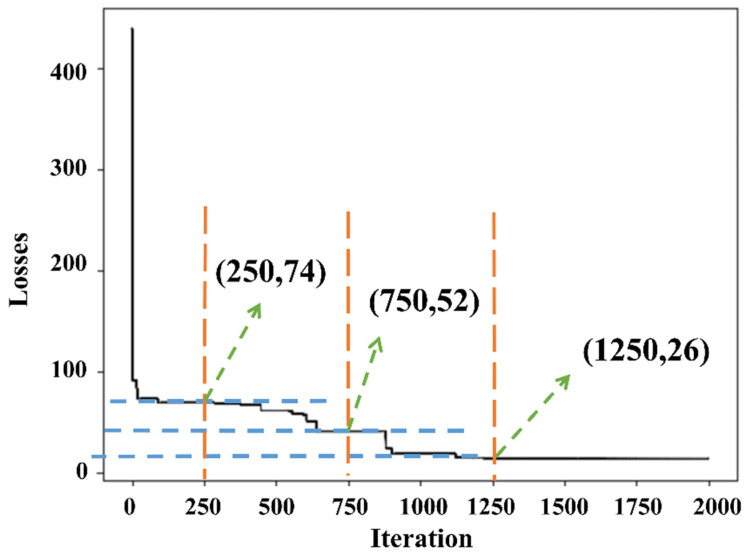
Change in loss value in iteration.

In addition to selecting neural network parameters, it is crucial to dynamically adjust model parameters during the training process to improve the performance and prediction accuracy of ANNs. After applying a GA optimization algorithm to optimize the parameters of the ANN, a population size of 8, parent mating at 4, 2000 generations, and a 5% mutation rate were chosen. To ensure that the GA converges to an optimal ANN structure and weight parameters, several techniques can be employed, including the selection process, crossover operation, mutation operation, fitness evaluation, and generation count. Random changes are introduced in the genetic material of individuals through mutation operations, which helps prevent premature convergence to suboptimal solutions. The genetically optimized ANN demonstrated a decreasing curve relationship between the loss function and the iteration number, indicating improved performance. This implies that as the number of iterations increases, the network loss gradually decreases, and the network fitting effect improves. Figure 6 shows the loss obtained via the genetically optimized ANN under different genetic generations, which can be used to measure the model performance. It is worth noting that different genetic generations can produce different results when optimizing the ANN through GA. In the initial phase of convergence, that is, the number of iterations is below 250. This is because the initial random population may produce better solutions due to genetic manipulation so that the loss value decreases rapidly. In the gradual convergence phase, as the algorithm continues to iterate, the optimization process may gradually approach the superior solution. At this point, the loss value may drop slowly in each iteration, around the number of iterations of 750, with a loss value of 52. The algorithm continuously improves in the search parameter space, utilizing better parameter combinations to improve the performance. As the number of iterations reaches 1250, the loss function converges to the local optimal solution. This means that while continuously optimizing the model, the loss function tends to stabilize at a loss value of 26, where the loss function is minimal, meaning the algorithm has converged and the neural network achieves better performance.

Finally, it is necessary to evaluate the model to continuously optimize the algorithm and improve its accuracy and performance in practice. Table 4 shows the statistical error analysis parameters comparison of the training dataset and test dataset. It is found that the optimized GA-ANN model has good results in training, with a correlation coefficient R^2^ greater than 0.95, and RMSE and MAE both less than 10, indicating a high fitting accuracy to the dataset. The conclusion shows that GA-ANN is suitable for predicting the strength performance of concrete with good accuracy.

### 3.2. Comparison of ANN and GA-ANN

**Figure 7:**
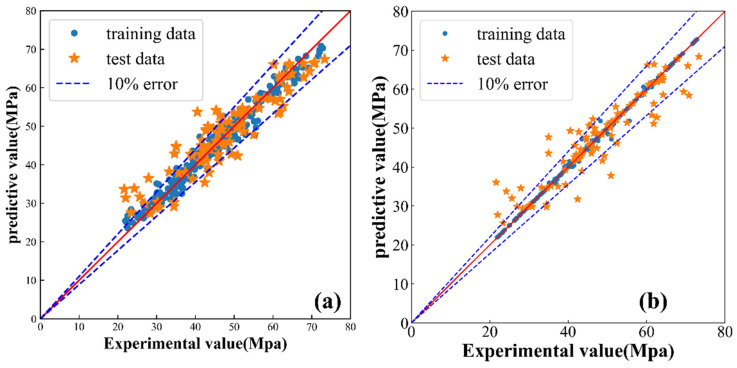
Comparison of ANN and ANN optimized via GA. (**a**) GA-ANN and (**b**) ANN.

The GA-ANN model was trained and evaluated by dividing the dataset into training, validation, and testing datasets. The error analysis shows that the GA-ANN model has excellent learning and fitting performance. To further verify the optimization effect of GA on the network structure, an ANN model with the same number of hidden layers and neurons was built to compare with GA-ANN. The prediction value is shown in Figure 7, and the loss value of the two models is listed in Table 5. For machine learning models, most of the errors between predicted values and experimental values were within the margin of ±10% [36], in which a large dispersion can be observed. Figure 7a shows the prediction results of the compressive strength for GA-ANN, which agrees well with the true value. GA-ANN achieves excellent testing results (R^2^ = 0.95) and the value of ANN (R^2^ = 0.80), indicating high accuracy in predicting concrete strength. These results suggest that the ANN model optimized by GA has better prediction accuracy than the ANN model alone, and the correlation coefficient R^2^ increases by about 15%. This finding demonstrates that GA can optimize the structure and parameters of neural network models and improve their prediction performance. GA searched for a better network structure by increasing, decreasing, or changing the connection mode between the layers and obtained the number of hidden layers in the neural network and the number of neurons. Moreover, GA can optimize the parameters of the neural network, but the resulting optimization result is usually a parameter combination rather than the visual graph structure, so it is difficult to show the details clearly. In summary, GA can improve prediction performance, providing an effective method for the practical application of concrete performance prediction.

### 3.3. Multi-Objective Optimization Based on Scipy

The purpose of multi-objective optimization is to find a set of solutions that optimize multiple interdependent objectives, ensuring that all objective functions are optimal or close to optimal [37,38]. However, the compressive strength of concrete often increases with the amount of cement, but this leads to increased cost, creating a contradiction. Previously, the GA-ANN model has been established between the amounts of raw materials and strength, which aims to minimize the prediction error and improve the performance in prediction tasks. In this chapter, the Scipy library will be used to establish two conflicting objective functions, material strength and material price, which aim to optimize the concrete mixture. Furthermore, it is crucial to analyze and evaluate the results of the optimized target based on the actual requirements.

To balance the need for improved concrete strength and lower cost, the Scipy library will be utilized for multi-objective optimization of concrete mixtures. First, two conflicting functions should be established, i.e., the target function of compressive strength and the content of each component raw material, which could be represented using the GA-ANN prediction model. By inputting the parameters of each experimental variable of the concrete into the established GA-ANN model, the compressive strength values with high accuracy were obtained. The target function for maximum compressive strength is briefly expressed in Equation (23). After collecting the information on raw materials, the relationship formula between the cost of eight experimental variables and the total price of concrete is established (as shown in Equation (25)), and the raw material prices are listed in Table 6. As a result, the target function for minimum cost of concrete is demonstrated in Equation (24).

After establishing two conflicting equations that require optimization, the range for eight variables was determined based on the basic range of concrete mix proportions. Boundary condition indicates whether the decision variables have a boundary (1) or not (0). The upper boundary is *B*_1_, and the lower boundary is *B*_2_. Table 7 shows the range for mix proportions and boundary conditions.

(23) $$f_{1}=max(GA-ANN(x_{1},x_{2},\ldots ,x_{8}))$$

(24) $$f_{2}=min\;(Cost\;(x_{1},x_{2},\ldots ,x_{8}))$$

(25) $$Cost\;(x)=0.4x_{1}+0.13x_{2}+0.37x_{3}+2x_{4}+0.135x_{5}+0.084x_{6}+0.0017x_{7}+3x_{8}$$

### 3.4. Precise Design of Concrete by GA-ANN and Spicy Models

#### 3.4.1. Development of Concrete Mixture Design Software

**Figure 8:**
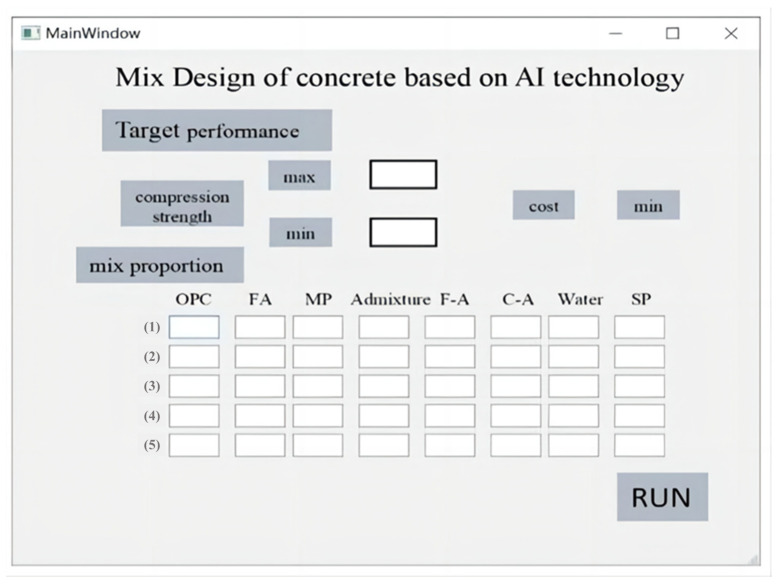
GUI of concrete mixture design.

In recent years, the rapid development of AI technology has greatly accelerated the process of intelligence and information in all walks of life and brought new opportunities and challenges to traditional industries [39,40]. In this study, a GA-ANN model combined with the Scipy model is developed for performance predicting and multi-objective design. However, despite the excellent performance of different kinds of AI technology ML models in concrete mixture design, it still has some problems in its practical application. In particular, the application of the GA-ANN and Scipy models is limited due to the lack of simple and efficient graphical user interface (GUI) software (V1.0). Therefore, based on the construction and research of GA-ANN and Scipy, this paper further builds a concrete mixture design system based on AI technology, as shown in Figure 8.

As the database grows larger and the complexity of ANN increases, computers need to have greater computing power, which is coordinated computing via a personal computer and a cloud computing server. The concrete mixture design system uses a GUI, which can obtain some concrete mixture design schemes, that satisfy the highest compressive strength and the lowest cost. The input module of the system includes cement (OPC), fly ash (FA), mineral powder (MP), other admixtures (AD), fine aggregate (F-A) and coarse aggregate (C-A), superplasticizer (SP), and water (W), and the output module is concrete mixture design schemes. The expert evaluation system provides great assistance and convenience for engineers and researchers because the performance prediction and the design results are based on the modeling of 487 tests in the database. The researcher can verify the test according to the mix ratio provided by the model and then adjust it according to the experience, reducing a lot of manual attempt time. In addition, through the design of GUI, researchers and engineers are also able to enrich the performance of concrete through the GA-ANN model and Scipy library, such as mobility, durability, toughness, etc., to better adapt to actual needs, and will conduct more in-depth research and exploration in the future.

#### 3.4.2. Multi-Objective Optimization of Concrete

In this study, to realize precise mixture design of concrete, a new software used for hybrid design method combining GA-ANN and Scipy optimization algorithm is proposed. The specific process of this method is described as follows:

(1) Material preparation: select the appropriate raw materials to determine their particle size distribution.
(2) Mixing design: determine the appropriate boundary conditions for raw material content, input the preliminary mixture into GA-ANN software (V1.0) for performance prediction, and then determine the design objectives and requirements (such as compressive strength of target range, minimum cost, etc.), and finally run the five sets of preliminary mixing ratios in the program.
(3) Expert review: evaluate whether the preliminary mixture meets the objectives and requirements, and score the five groups of mix ratios. If the requirements are not met, repeat steps (2) to adjust until the objectives and requirements are met to obtain the optimized mixture.
(4) Sample preparation: the concrete is prepared according to the optimized mixture ratio, and the feasibility of the mixture is further verified.

Furthermore, to demonstrate the feasibility and utility of this hybrid design approach, the following is a schematic diagram of the application of specific hybrid design examples.

**Figure 9:**
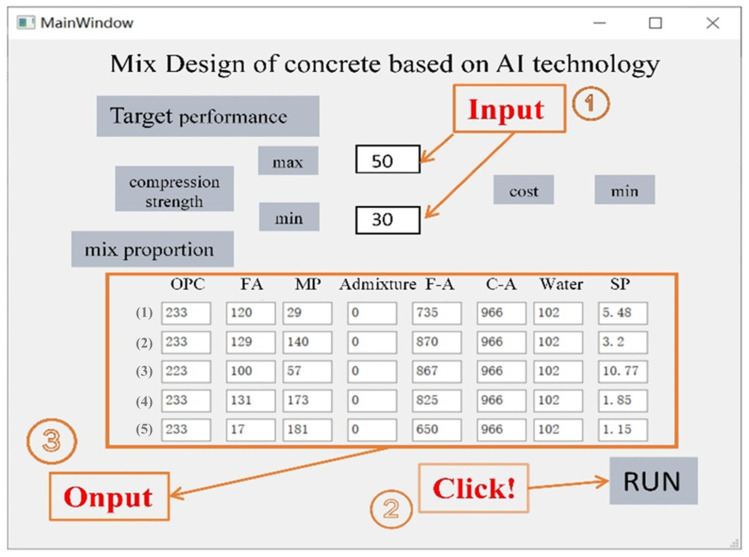
Software process of concrete mixture design.

During the process (2) mixing design, the concrete mix design software operates as shown in Figure 9. Firstly, the upper and lower limits of strength are confirmed according to the engineering requirements. For example, in the operating case, the input range is a minimum strength of 30 MPa and a maximum strength of 50 MPa. In practical engineering applications, a lower production cost is often preferred. Therefore, the software defaults to the minimum value of the price function. Click the ‘RUN’ button to start the mixture design, and the waiting time is only a few seconds. In the third step, the software automatically generates five sets of mixture design schemes, which are the top five mix ratios that meet the concrete strength range requirements and have the lowest price.

During the process (3), it is necessary to rely on specific project requirements to carry out expert evaluation. First, the strength of the five mixed design proportions obtained in the process are tested (2). Then, the price of concrete per cubic meter of sample is calculated based on Equation (25). Expert evaluation is a scoring system based on the target performance requirements. Below are three examples of expert evaluation scoring:

Example 1: Single objective—maximum compressive strength

The single target design follows the principle of highest intensity. Within the software, after entering the target strength range and running it, the output mix proportion data are displayed in groups, ranked from high to low according to strength. The mixture with the highest strength scores 10 points, and each lower rank reduces by two points, with the mix with the lowest strength scoring 0 points. The first set of data obtained is the mixed proportion data with the highest design strength and can be easily selected as the single target design. For example, in Table 8, the first group is designed with 10 points, and the fifth group is 0 points, so the first group is selected as the target design.

Example 2: Single objective—minimum economic cost

The single target design follows the principle of minimum cost. Use the economic cost principle and Equation (22) for price calculation. After ranking the prices from high to low, apply the same scoring criteria as in the intensity principle. Then, select the mix proportion with the lowest price per cubic meter of concrete as the target design mix proportion. For instance, in Table 8, the first set of mix proportions has the lowest price and a score of 10 points, making it the ideal target design of mix proportions.

Example 3: Multi-objective

The multi-objective design of fixed-strength range and economic cost. Obtain five sets of concrete mix designs within the target strength range and rank them based on both strength and price in descending order, using the same scoring method as mentioned above. The final score is obtained by adding up the scores. For example, in Table 8, the first set of designs scores 10 points for strength and 10 points for economic cost, resulting in a total score of 20 points. As the first design scheme has the highest score, it is chosen as the optimized experimental scheme. As the validation values shown in Table 8, the accuracy of the target compressive strength reached 97.3%. It is proved that the obtained target compressive strength is basically consistent with the experimental value, and the multi-objective design model is reliable.

Consequently, Scipy offers several advantages: versatility, ease of use, and performance, which provide a user-friendly interface and comprehensive documentation, making it relatively easy for researchers and engineers to implement and apply multi-objective optimization. Scipy is known for its efficient and robust implementation of optimization algorithms. It leverages optimized numerical routines and algorithms, resulting in faster convergence and improved computational efficiency. Hybrid models are highly flexible and scalable for various multi-objective solving problems. Using both ANN expression ability and Scipy’s optimization algorithm, multiple objective functions were established, and a set of balanced optimal solutions were found. It provides decisionmakers the ability to make trade-offs between different goals and obtain good solutions, which improves efficiency and accuracy. That is the reason why the hybrid methods can optimize not only a single target of compressive strength but also the design for both cost and strength.

## 4. Conclusions

This article introduces a novel method for designing concrete mixes, which utilizes ANN, GA, and Scipy library for hybrid intelligent modeling. By predicting the mechanical properties of concrete, this method can also establish objective functions that effectively optimize the single or multi-objective proportion design of specific mixtures.

(1) Genetic algorithms (GA) can optimize ANN structure and weight parameters, improving prediction accuracy and generalization. The results show a 19% increase in correlation coefficient (R^2^) compared to ANN. GA-ANN achieves excellent training results (R^2^ > 0.95, RMSE and MAE < 10), indicating high accuracy in predicting concrete strength.
(2) To balance the need for higher concrete compressive strength and lower cost, the Scipy library was utilized for the multi-objective optimization of concrete mixture. The multi-objective design framework mainly includes two parts: determine the range for ingredients proportions and boundary conditions and establish two conflicting functions, including the target function of strength and the cost of concrete. Through the experimental validation, the accuracy of the model reached 97.3%, and the best proportion reached 46.3 MPa, which met both the compressive strength and the low-cost requirements. It is proved that the obtained target compressive strength is basically consistent with the experimental value,
(3) Based on the established ML models, this paper further builds a concrete mixture design graphical user interface (GUI) software (V1.0), which not only can predict the compressive strength of concrete but also provide reliable guidance for researchers and engineers to concrete mixture design.
(4) This study introduces an innovative method that integrates artificial intelligence technology into concrete research, allowing for multi-objective design and property prediction. By combining AI with concrete technology, it maximizes the information processing capabilities in the concrete industry. However, further research is needed to enhance the interpretability, accuracy, and generalization of artificial neural networks. This will contribute to future advancements in the field of concrete and the continued development of AI applications.

## Figures and Tables

**Table 1:** Characteristics of cementitious material.

Cement		Mineral Powder		Fly Ash		
Density (g/cm^3^)	Specific Surface Area (m^2^/g)	Activity Index (%)	Specific Surface Area (m^2^/g)	Activity Index (%)	Density (g/cm^3^)	45 μm Sieve Residue
2.98–3.16	304–414	95–122	401–576	71–97	2.25–2.58	2.5–29.2

**Table 2:** Characteristics of aggregate.

Void Ratio of Coarse Aggregate (%)			Fineness Modulus of Fine Aggregate	
Gravel 5–10 mm	Gravel 10–20 mm	Gravel 16–31.5 mm	Machine-Made Sand	Natural Sand
37–45	38–45	42–44	2.4–3.3	2.5–3.1

**Table 3:** Description of the input and output data of the predicted intensity.

Variable	Symbol	Category	Statistics			
			Min	Max	Average	STDEV
Cement	OPC	input	174	494	309	70.7
Fly ash	FA	input	0	137	64	37.4
Mineral powder	MP	input	0	182	14.3	31.2
Admixtures	Admixture	input	0	34	0.1	2.1
Fine aggregate	F-A	input	635	935	773	54.1
Coarse aggregate	C-A	input	996	1241	1082.6	40.5
Water	Water	input	102	185	156.3	7.5
Superplasticizer	SP	input	0	12.8	4.2	1.6
Compressive Strength	T_1_	MPa	output	21.6	73.3	45.8
Cost	T_2_	¥/kg	output	292.87	405.71	345.66

**Table 4:** Statistical error analysis parameters for GA-ANN.

Training Data				Testing Data			
R^2^	MSE	RMSE	MAE	R^2^	MSE	RMSE	MAE
0.96	60.91	7.80	5.37	0.95	61.94	7.87	5.45

**Table 5:** Comparison of model parameters between GA-ANN and ANN.

Model	R^2^	MSE	RMSE	MAE
GA-ANN	0.95	61.94	7.87	5.45
ANN	0.80	82.74	9.09	3.61

**Table 6:** Prices of raw materials in Mainland China.

Component		Units	Cost (¥)
Cement	*x*_1_	kg	0.4
Fly ash	*x*_2_	kg	0.13
Mineral powder	*x*_3_	kg	0.37
Admixtures	*x*_4_	kg	2.00
Fine aggregate	*x*_5_	kg	0.135
Coarse aggregate	*x*_6_	kg	0.084
Compound superplasticizer	*x*_7_	kg	3
Water	*x*_8_	kg	0.0017

**Table 7:** Range and boundary of variables.

	Variety	OPC	FA	MP	AD	F-A	C-A	W	SP
Range (kg)	Min	180	0	0	0	600	1000	140	0
	Max	500	130	200	50	900	1200	180	10
Boundary	B_1_	1	1	1	1	1	1	1	1
	B_2_	1	1	1	1	1	1	1	1

**Table 8:** Cost of five designed mixtures.

Varieties	OPC (kg)	FA (kg)	MP (kg)	AD (kg)	F-A (kg)	C-A (kg)	Water (kg)	SP (kg)	Strength (MPa)	Validation (MPa)	Cost (¥)
1	233	120	29	0	735	966	102	5.48	47	46.3	316.51
2	233	129	140	0	870	966	102	3.2	45	45.5	370.14
3	233	100	57	0	867	966	102	10.77	45	44.7	357.96
4	233	131	173	0	825	966	102	1.85	41	42.1	372.48
5	233	17	181	0	650	966	102	1.15	39	38.6	334.90

## Data Availability

Not applicable.
